# The application of nitrate nitrogen fertilizer reduces the occurrence of bacterial fruit blotch of watermelon

**DOI:** 10.3389/fpls.2026.1869653

**Published:** 2026-07-08

**Authors:** Dehua Liu, Mei Zhao, Pei Qiao, Weiqin Ji, Wenjun Zhao, Tingchang Zhao, Wei Guan, Yuwen Yang, Lulu Cai

**Affiliations:** 1 Anhui Jianghuai Horticulture Seed Industry Co., Ltd., Hefei, China; 2 State Key Laboratory for Biology of Plant Diseases and Insect Pests, Institute of Plant Protection, Chinese Academy of Agricultural Sciences, Beijing, China; 3 Department of Plant Pathology, College of Plant Protection, China Agricultural University, Beijing, China; 4 Chinese Academy of Quality and Inspection & Testing, Beijing, China

**Keywords:** *Acidovorax citrulli*, bacterial fruit blotch, *Citrullus lanatus*, disease resistance, nitrogen form

## Abstract

Bacterial fruit blotch (BFB), caused by *Acidovorax citrulli*, is a devastating seed-borne disease of cucurbit crops. The chemical form of nitrogen fertilizer is a key factor modulating host plant defense and pathogen virulence. In this study, we investigated the effects of three nitrogen regimes on watermelon resistance to BFB by assessing disease severity, seedling biomass, stem vascular morphology, expression of defense-related genes, and *in planta* growth of *A. citrulli*. Our results showed that sole ammonium nitrogen application led to a significantly higher disease index compared with nitrate nitrogen alone or a 1:1 nitrate-ammonium mixture. Moreover, nitrate nitrogen application enhanced watermelon seedling growth, calcium uptake, development of stem vascular tissue, and expression of disease resistance-related genes. Temporal dynamics revealed that *A. citrulli* populations in cotyledons were initially lower under sole ammonium but surpassed nitrate and mixed nitrogen treatments at later infection stages. Furthermore, the expression of type III secretion system genes *hrpG* and *hrpE* in *A. citrulli* from watermelon cotyledons initially decreased and then increased over time, with this fluctuating expression pattern accelerating as the ammonium proportion in the fertilizer increased. This finding suggests that *A. citrulli* establishes infection more rapidly in watermelon seedlings under sole application of ammonium nitrogen. Together, nitrate nitrogen mitigates BFB severity in watermelon, whereas sole ammonium exacerbates disease likely via combined effects of weakened plant vigor, altered tissue nutrition, and accelerated pathogen infection. This study offers a theoretical foundation for nutrient−based management of watermelon BFB.

## 1 Introduction

Bacterial fruit blotch (BFB), caused by *Acidovorax citrulli*, is a destructive seed-borne disease that causes severe economic losses to cucurbit crops, including watermelon (*Citrullus lanatus*) and melon (*Cucumis melo*) (Zhao and Walcott, 2018). First documented in Georgia, USA, in 1965 (Webb and Goth, 1965), BFB has since spread globally, with reports in numerous countries, posing a persistent and serious threat to global cucurbit production industry (Zhao and Walcott, 2018). To date, the most effective management strategy for BFB remains the prevention of planting infected seeds and seedlings, as this measure directly interrupts the primary transmission route of the pathogen (Burdman and Walcott, 2012).

Nitrogen is an essential macronutrient for plant growth and development, accounting for approximately 1.5-2% of plant dry matter and 16% of total plant protein content. Beyond sustaining basic plant physiological processes, nitrogen is a core component of proteins and various bioactive compounds that mediate plant defense responses against pathogens (Scheible et al., 2004, Héctor and San Segundo, 2025). Through primary and secondary nitrogen metabolism, plants modulate their cellular structure and biochemical composition. These changes directly affect the thickness of physical defense barriers (e.g., cell walls, cuticles), thereby enhancing innate disease resistance (Plavcová et al., 2013; Camargo et al., 2014). Notably, different chemical forms of nitrogen nutrition exert distinct effects on the physical defense capabilities of plants. For example, compared with nitrate (NO_3_^-^)-based nutrition, amino acid-based nitrogen nutrition improves the quality of epidermal wax on cabbage leaves (Blanke et al., 1996). Conversely, ammonium (NH_4_^+^) has been shown to boost the activity of peroxidase isoenzymes, which are key enzymes involved in lignin synthesis, thereby strengthening cell wall structure and enhancing physical resistance to pathogen invasion (Wang et al., 2010).

In addition to regulating physical defense systems, nitrogen mediates a spectrum of chemical defense mechanisms in plants. This includes the biosynthesis of defensive plant metabolites (e.g., phytoalexins, antibacterial proteins, amino acids, organic acids) and the activation of defense-related enzymes, both of which contribute to inhibiting pathogen growth and mitigating infection (Wang et al., 2019; Zeier, 2013; Ngadze et al., 2012). The nitrate (NO_3_^-^) to ammonium (NH_4_^+^) ratio in the growth medium further modulates the production of carbon-based secondary metabolites (e.g., ginsenosides, artemisinin) (Zhang et al., 1996; Wang and Tan, 2002; Zhang et al., 2011). The stimulatory effect of NO_3_^-^ on secondary metabolite biosynthesis is thought to be linked to defense signaling pathways involving nitric oxide (NO), which is derived from nitrate reductase activity (Thapa et al., 2018; González-Hernández et al., 2019; Sarhan et al., 1982). Furthermore, nitrogen can enhance the activity of antioxidant enzymes, thereby improving antioxidant capacity, reducing oxidative damage to cell membranes, and maintaining cellular integrity under biotic stress (Yao and Liu, 2007; Campos et al., 2019).

At the molecular level, different nitrogen forms also play divergent roles in modulating plant resistance to pathogens. Ammonium (NH_4_^+^)-mediated plant immunity is closely associated with amino acid metabolism. Changes in amino acid pools can either restrict pathogen nutrient acquisition or alter the expression of defense-related genes, thereby influencing disease outcomes (Seifi et al., 2013). In contrast, nitrate (NO_3_^-^) nutrition enhances downstream immune responses through NO-dependent signaling pathways, which amplify the expression of defense-related genes and enhance overall disease resistance (Wang et al., 2013).

Zimerman-Lax et al. (2016) conducted a pivotal study to elucidate the effects of different nitrogen fertilizer forms on BFB development in melon seedlings. Their results showed that exclusive application of nitrate nitrogen reduced the population of *A. citrulli* in melon leaves and alleviated BFB severity compared to the mixed application of ammonium and nitrate nitrogen. Additionally, melon seedlings treated with nitrate nitrogen alone exhibited significantly higher leaf calcium content, a nutrient known to reinforce cell walls and enhance disease resistance. Gene expression analysis revealed that, following inoculation with *A. citrulli*, the expression of the gene encoding hydroperoxide lyase (HPL, an enzyme involved in lipid metabolism and defense signaling), was significantly downregulated in melon seedlings treated with nitrate nitrogen alone. In contrast, the genes encoding plant defensin (PDF1.4, a secreted antimicrobial peptide) and peroxidase 34 (PRX34, an antioxidant enzyme) were significantly upregulated in all treated melon seedlings after inoculation, with no consistent regulatory effect of nitrogen fertilizer forms on the expression of these two genes (Zimerman-Lax et al., 2016).

To date, among the characterized watermelon defense-related genes, *PLATZ6*, a member of the PLATZ family, encodes a novel DNA-binding protein associated with plant growth, development, and stress responses in rice, watermelon, and *Arabidopsis thaliana*. Heterologous overexpression of watermelon *PLATZ6* in *Arabidopsis* has been shown to significantly inhibit the proliferation of *Pseudomonas syringae* pv. *tomato* in *Arabidopsis* (Qi, 2021). Additionally, defensin (PDF2.4) and callose synthase (CALS7) have been shown to play important roles in plant resistance to pathogens (Kong et al., 2017; Qi, 2021; Xie et al., 2011).

## 2 Materials and methods

### 2.1 Plant materials, nutrient solutions, and bacterial strains

The watermelon cultivar ‘Ruixin’, provided by the Institute of Vegetables and Flowers, Chinese Academy of Agricultural Sciences (Beijing, China), was used in this study. Seeds were sown in vermiculite, and seedlings were grown in a controlled-environment growth chamber under the following conditions: 14 h light at 26 °C, 10 h dark at 20 °C, and 60% relative humidity. Seedlings were irrigated with nutrient solutions differing in nitrogen form (**Table 1**) supplemented with a water-soluble trace element fertilizer (Sichuan Guoguang Agrochemical Co., Ltd., China). During the entire cultivation period, leachate from the vermiculite substrate was regularly collected and monitored for pH and electrical conductivity (EC). Dilute sulfuric acid or sodium hydroxide solution was used to adjust the pH to 6.0, and distilled water was applied to adjust the EC to 2.8 mS/cm. These real−time dynamic adjustments ensured consistent pH and EC values across all treatments throughout the experiment.

**Table 1:** Composition of nutrient solutions with different nitrogen fertilizer forms (mmol/L).

Material	N1 (sole nitrate nitrogen)	N2 (1:1 mixture of nitrate and ammonium nitrogen)	N3 (sole ammonium nitrogen)
KNO_3_	10.0	5.0	0
Ca(NO_3_)2	10.0	5.0	0
CaCl_2_	0	5.0	10.0
(NH_4_)_2_SO_4_	0	7.5	15.0
KH_2_PO_4_	4.0	4.0	4.0
KCl	0	5.0	10.0

To evaluate the impact of nitrogen nutrition on disease severity, three nutrient solutions with a uniform nitrogen concentration (30 mmol/L) but differing in nitrogen forms were prepared: N1 (sole nitrate nitrogen), N2 (1:1 mixture of nitrate and ammonium nitrogen), and N3 (sole ammonium nitrogen). The specific components and their final concentrations in each nutrient solution are detailed in **Table 1** (Zimerman-Lax et al., 2016). All nutrient solutions were freshly prepared prior to each application to prevent precipitation.

All experiments used *Acidovorax citrulli* strain Aac5, a naturally ampicillin (Amp)-resistant strain. The bacterium was cultured at 28° C in King’s B (KB) medium (tryptone 20 g, MgSO_4_·7H_2_O 1.5 g, K_2_HPO_4_ 1.5 g, glycerol 15 mL, and 1000 mL deionized water). The working concentration of ampicillin was 100 μg/mL.

### 2.2 Spray inoculation assay

Overnight Aac5 cultures were harvested, and bacterial density was adjusted to an optical density at 600 nm (OD_600_) of 0.3 with sterile water. Each treatment included four watermelon seedlings at the three- to four-true-leaf stage, with each seedling sprayed with 10 mL of the bacterial suspension or sterile water (negative control). To maintain high relative humidity, inoculated seedlings were enclosed in plastic bags immediately after treatment, then transferred to a growth chamber set to a 16 h light period at 28° C and an 8 h dark period at 22° C, with 80% relative humidity. Disease severity was evaluated at 14 days post inoculation (dpi) using a 0–6 rating scale (Ofir et al., 2009): 0 = no symptoms; 1 = ≤10% symptomatic leaves; 2 = 11–25% symptomatic leaves; 3 = 26–50% symptomatic leaves; 4 = 51–75% symptomatic leaves; 5 = 76–90% symptomatic leaves; 6 = >90% symptomatic leaves. This experiment was conducted three times.

### 2.3 Determination of bacterial growth in watermelon cotyledons

To assess *in planta* growth of *A. citrulli* strain Aac5 in watermelon cotyledons, a bacterial suspension was prepared to a concentration of 10^6^ colony-forming units (cfu/mL) with sterile water. The suspension was injected into the cotyledons using a 1 mL syringe, with sterile water infiltration as the negative control. Seedlings were then incubated in a growth chamber under the same conditions as described above. At designated time points (1, 24, 48, 72, 96, 120, and 168 h post inoculation (hpi)), watermelon cotyledons were randomly sampled for observation and imaging. For each treatment, nine seedlings were sampled, with one cotyledon collected per seedling, yielding a total of nine cotyledons per treatment. Three leaf discs (0.8 cm in diameter) were excised from each cotyledon and homogenized with 500 μL sterile water in a 2 mL microcentrifuge tube. The resulting homogenates were subjected to ten-fold serial dilution, and 10 μL aliquots of each dilution were plated onto KB agar supplemented with Amp. Plates were incubated at 28 °C for 48 h, and colony counts were subsequently recorded (Ren et al., 2014). This experiment was conducted three times.

### 2.4 Determination of bacterial growth in the stem

To assess the *in planta* bacterial growth of *A. citrulli* in watermelon stems, seeds were sown in vermiculite and cultivated with N1, N2, and N3 nutrient solutions until reaching the three-true-leaf stage. Each treatment included three biological replicates, with six seedlings per replicate. A bacterial suspension of *A. citrulli* strain Aac5 (10^8^ cfu/mL) was inoculated into the stem near the cotyledons by needle pricking using a 1 mL syringe. Stem samples were collected at 10 days post-inoculation. For bacterial population quantification, a 3-cm stem section adjacent to the cotyledons was excised, placed in a microcentrifuge tube containing 500 μL of sterile water, and thoroughly homogenized. A 10 μL aliquot of the homogenate was plated on KB agar supplemented with Amp, followed by incubation at 28° C for 48 h. Bacterial colonies were then enumerated to determine the bacterial population density.

### 2.5 Determination of biomass and nutrient element contents in watermelon seedlings

The biomass of watermelon seedlings was assessed by measuring plant height, root length, stem diameter, fresh weight, and number of leaves. Sampled seedlings were thoroughly rinsed three times with tap water and once with deionized water, then oven-dried at 105 °C for 30 minutes, followed by further drying at 80 °C for 5 h to a constant weight for dry weight determination. Each treatment included ten replicates for biomass measurements. For nutrient analysis, mature leaves and main stems were collected from the treated seedlings, and the samples were sent to Nanjing Origin Testing Technology Co., Ltd. (China) for the quantification of nitrogen, phosphorus, potassium, and calcium concentrations. The experiment was conducted three times.

### 2.6 Determination of the *A. citrulli* growth in watermelon leaf and stem extracts

Watermelon seedlings were grown for five weeks with nutrient solutions containing different nitrogen forms (N1, N2, N3). Stems and leaves of seedlings from each treatment were collected, finely sectioned, and 10 g of the tissue was weighed and placed into a mortar. Then 10 mL of sterile deionized water was added, and the mixture was thoroughly ground to complete homogenization. The homogenate was filtered through sterile gauze and the filtrate was collected in a 15 mL centrifuge tube. The tube was incubated at room temperature for 1 h to facilitate sedimentation, then centrifuged at 7000 rpm for 3 minutes. The supernatant was filtered through a sterile 0.22 μm nitrocellulose membrane to obtain sterile leaf and stem extracts of watermelon.

A suspension of *A. citrulli* Aac5 was prepared with sterile water and adjusted to an OD_600_ of 0.3. Subsequently, 10 μL of the bacterial suspension was mixed with 990 μL of the sterile leaf or stem extract from each nitrogen treatment. A 200 μL aliquot of each diluted bacterial mixture was transferred to 100-well polystyrene plates and incubated at 28 °C with continuous shaking at 220 rpm using a Bioscreen C PRO instrument (Finland). The OD_600_ values were measured every 2 h for 72 h (Liu et al., 2023). Each treatment included four replicates, and the experiment was conducted at least three times.

### 2.7 Histological analysis of watermelon seedling stems

Watermelon seedlings were grown for two weeks with nutrient solutions of different nitrogen forms (N1, N2, N3), then spray inoculated with *A. citrulli* strain Aac5. After an additional two weeks of cultivation, stem phloem tissues were sectioned and observed microscopically. Four-week-old non-inoculated watermelon seedlings were used as controls. For sectioning, 1-cm stem segments were excised with a sterile blade. Thin, flat transverse sections were prepared and mounted on glass slides. Subsequently, 50 μL of lignin acidification solution was added and left for 2 minutes, followed by the addition of 50 μL of phloroglucinol staining solution. After another 2 minutes, a coverslip was placed over the section. The thickness of the sieve tube cell walls in the phloem was measured via microscopic observation and imaging. For each treatment, five seedlings were randomly selected, and the cell wall thickness of 50 sieve tube elements was measured per seedling. The experiment was conducted at least three times.

### 2.8 Determination of expression levels of defense-related genes in watermelon

Overnight cultures of *A. citrulli* strain Aac5 were harvested and adjusted to an OD_600_ of 0.3 with sterile water. The bacterial suspension was then diluted 100-fold with sterile water and infiltrated into the cotyledons and first true leaf of 3-week-old watermelon seedlings pretreated with different nitrogen forms, using a 1 mL needleless syringe. Inoculated seedlings were incubated in a growth chamber and samples were collected at 0, 24, 48, and 72 hours post-inoculation (hpi). Total RNA was extracted from seedling tissues using a plant total RNA extraction kit (Yisheng Biotechnology, China). RNA concentration was quantified, and RNA samples were stored at -80 °C for subsequent experiments. First-strand cDNA was synthesized using the FastKing gDNA Dispatching RT Supermix Kit (TIANGEN, China) according to the manufacturer’s instructions.

Quantitative real-time PCR (qRT-PCR) was performed using the relative quantification method (Kong et al., 2017), with *CICAC* and *CITUA* serving as internal reference genes for normalization. The expression level of each target gene in N1-treated (sole nitrate nitrogen) seedlings at 0 hpi was set as the calibrator (value=1). Relative expression levels at 0, 1, 2, and 3 dpi under different nitrogen treatments were calculated based on cycle threshold (Ct) values.

Four watermelon defense-related genes (*PLATZ6*, *HPL*, *PDF2.4*, and *CALS7*) were selected for expression analysis based on the CuGenDB database (http://cucurbitgenomics.org/v2/). Gene-specific primers for qRT-PCR were designed using Primer 5.0 software. Primer sequences and expected amplicon lengths are listed in **Table 2**.

**Table 2:** Primers used for qRT-PCR analysis of defense-related genes in watermelon.

Primers	Primer sequence (5’-3’)	Length (bp)
CIPLATZ6-F	CCAGCCCAGAAACAAATGTAAGC	107
CIPLATZ6-R	TCCGCGTTTTCTTGGTTGTG	
CIHPL-F	CATCGCCTCTCGCTACCAA	145
CIHPL-R	ACCGTAGGCGTTGAATCCC	
CIPDF2.4-F	TTTTGGCACTGGGATGGAG	179
CIPDF2.4-R	CGCAGTGCTTTGTGCAGAA	
CICALS7-F	TGCCAAGGAGGTTCAGAGGA	180
CICALS7-R	TCATCGGGATTGCTGGCT	
CICAC-F	AATTGTGGTTGATGCTGCAC	94
CICAC-R	TGACAGCTGTACCTGGCATC	
CITUA-F	CTTGCTGGGAGCTCTATTGC	105
CITUA-R	AACGGATTAAAAGCGTCGTG	

### 2.9 Determination of the expression levels of virulence-related genes in *A. citrulli*

*Acidovorax citrulli* strain Aac5 was cultured in KB medium. Total RNA was extracted from the bacterial cells using a bacterial total RNA extraction kit (Yeasen, China). A bacterial suspension was prepared and adjusted to an OD_600_ of 0.3 with sterile water. This suspension was then diluted ten-fold with sterile water and infiltrated into the cotyledons of 3-week-old nitrogen-pretreated watermelon seedlings using a 1 mL needleless syringe. Inoculated seedlings were maintained in a growth chamber and sampled at 24, 48, and 72 hpi. Total RNA was extracted from infected tissues using a bacterial total RNA extraction kit (Yisheng Biotechnology, China) and cDNA was synthesized using the FastQuant RT Kit (Yeasen, China). For gene expression analysis, the gene *rpoB* was used as the internal reference. Target genes included *ntrB* and *ntrC* (nitrogen metabolism regulation), *hrpG* and *hrpE* (type III secretion system, T3SS), *flhD* and *fliA* (flagellar assembly), and *pilA* (pilin subunit). qRT-PCR was performed to determine their expression levels. Primer sequences used in the assay are listed in **Table 3**. The average expression level of each target gene in *A. citrulli* Aac5 at 0 hpi was set to 1.0 for normalization. Relative expression was calculated using previously reported methods (Jin et al., 2004; Gao et al., 2011; Livak and Schmittgen, 2001). All experiments were performed three times independently.

**Table 3:** Primers for virulence-related and internal reference genes in *Acidovorax citrulli*.

Primers	Primer sequence (5’-3’)	Length (bp)
rpoB-F	GCGACAGCGTGCTCAAAGTG	134
rpoB-R	GCCTTCGTTGGTGCGTTTCT	
ntrB-qF	GGGCTGGGACTGACGCTT	110
ntrB-qR	CAGGGCAAGGGAATGAGGA	
ntrC-qF	GGCCTGCCGGTCATCATC	158
ntrC-qR	ACCTCTTCCCGCTGGCTTT	
flhD-F	TTCGTTGCCGACCTTCTTG	171
flhD-R	TTCCGCCTGGGCATCAA	
fliA-F	ACACCGCCAAAGGACAGCT	178
fliA-R	CCCTGCGTCACCTCGTAGC	
hrpG-F	CTCGCCTGGCTGCTGTT	197
hrpG-R	GCTTGTAGATGTGCTGCTCC	
hrpE-F	GTCAGGATGGACACGCAGGC	119
hrpE-R	AACGCATGGTGCTGGCAGAG	
pilA-F	TCAAGACGACTGCCGATGG	150
pilA-R	AAGAGCCAACTTGCTGCCC	

### 2.10 Statistical analyses

Experimental data were compiled and analyzed using Microsoft Excel 2016 (Microsoft, USA). Statistical analyses were performed using GraphPad Prism 7 (GraphPad Software, USA). One-way analysis of variance (ANOVA) was used to assess significant differences among different nitrogen treatments for all measured indices in watermelon seedlings. For sieve tube cell wall thickness in watermelon stems, independent-samples t-tests were used to determine significant differences between inoculated and non-inoculated groups within the same nitrogen treatment.

## 3 Results

### 3.1 Effects of nitrogen nutrition on disease severity

**Figure 1:**
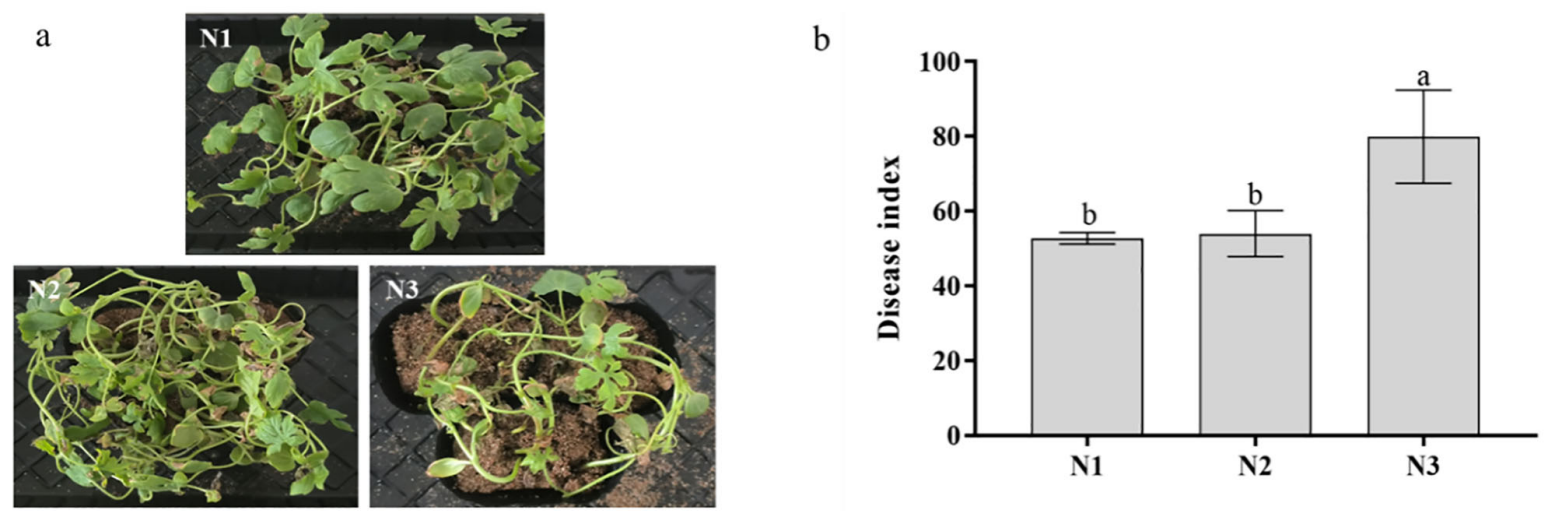
Evaluation of bacterial fruit blotch via spray inoculation. **(a)** Symptoms of watermelon seedlings at 14 days after spray inoculation with *Acidovorax citrulli* under different nitrogen fertilizer treatments; **(b)** Disease index of watermelon seedlings under N1 (sole nitrate nitrogen application), N2 (1:1 mixed nitrate and ammonium nitrogen), and N3 (sole ammonium nitrogen). Different lowercase letters indicate significant differences among treatments (one-way ANOVA, *P* < 0.05).

To evaluate the effect of different nitrogen forms on BFB severity, spray inoculation was conducted after the application of nitrogen fertilizer. At 14 dpi, watermelon seedlings under the N3 treatment (sole ammonium nitrogen) displayed the most severe BFB symptoms. The disease indices for seedlings under N1 (sole nitrate nitrogen), N2 (1:1 mixed nitrate and ammonium nitrogen), and N3 treatments were 52.75, 53.96, and 79.92, respectively. The disease index of the N3 treatment was significantly higher than those of the N1 and N2 treatments (**Figure 1**).

### 3.2 Bacterial growth *in planta* of *A. citrulli* Aac5 in watermelon cotyledons

**Figure 2:**
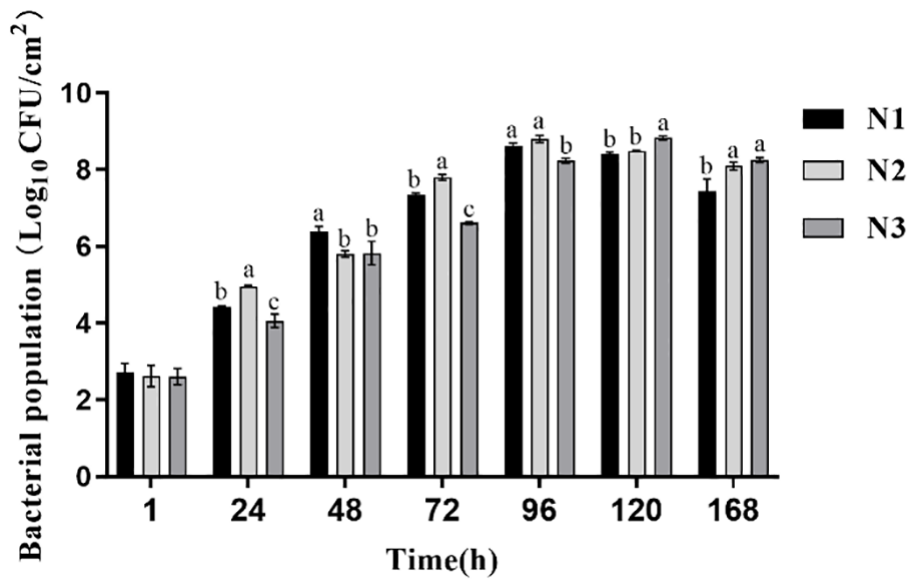
*In planta* growth of *Acidovorax citrulli* Aac5 in watermelon cotyledons treated with different nitrogen forms: N1 (sole nitrate nitrogen), N2 (1:1 mixed nitrate and ammonium nitrogen), and N3 (sole ammonium nitrogen). Bacterial population levels were measured in watermelon cotyledons inoculated with the tested strain at 1, 24, 48, 72, 96, 120, and 168 h post-inoculation (hpi). Error bars represent the standard errors of the means. Different lowercase letters at the same time point indicate significant differences (one-way ANOVA, *P* < 0.05).

No significant differences in the timing of symptom onset or severity were observed among treatments; however, quantification of bacterial populations in cotyledons revealed a distinct temporal colonization dynamic. Prior to 96 hpi, the bacterial populations in N3-treated watermelon cotyledons were significantly lower than those in N1- and N2-treated cotyledons. Interestingly, from 120 hpi onwards, the bacterial populations in N3-treated cotyledons became significantly higher than those in N1 and N2 treatments (**Figure 2**).

### 3.3 *In planta* growth of *A. citrulli* Aac5 in watermelon stems

**Figure 3:**
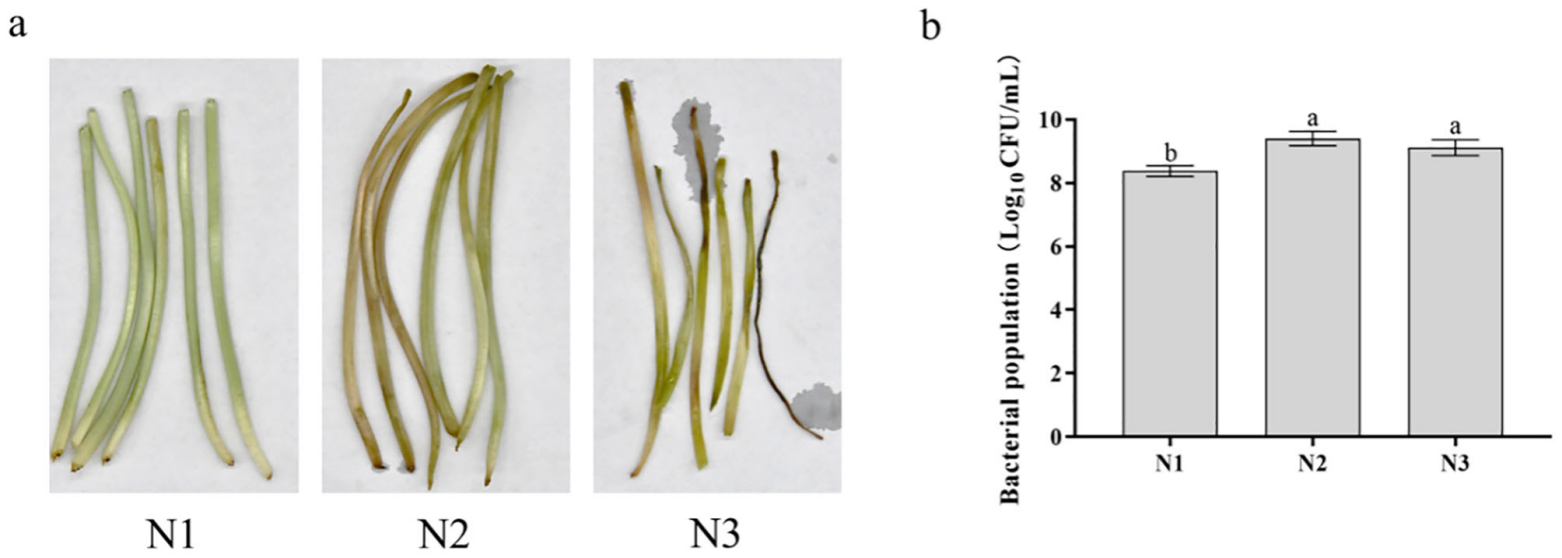
*In planta* growth of *Acidovorax citrulli* Aac5 in watermelon stems under different nitrogen fertilizer treatments. **(a)** Symptoms of watermelon stems treated with N1 (sole nitrate nitrogen), N2 (1:1 mixed nitrate and ammonium), and N3 (sole ammonium nitrogen) at 10 days after inoculation with Aac5; **(b)** Viable bacterial counts in stem segments. Error bars represent the standard errors of the means. Different letters indicate significant differences among treatments (one-way ANOVA, *P* < 0.05).

Watermelon stems from plants grown with different nitrogen forms were inoculated with *A. citrulli* Aac5. After 10 days, samples were collected for observation. Stems treated solely with ammonium nitrogen (N3) displayed the most severe disease symptoms (**Figure 3a**). Viable bacterial counts in stem segments were quantified, revealing populations of 2.73 × 10^9^ cfu/mL and 1.43 × 10^9^ cfu/mL under the N2 and N3 treatments, respectively. Both N2 and N3 treatments supported significantly higher bacterial populations than the N1 treatment (2.50 × 10^8^ cfu/mL) (**Figure 3b**).

### 3.4 The effects of different nitrogen fertilizer treatments on biomass and nutrient elements of watermelon seedlings

**Figure 4:**
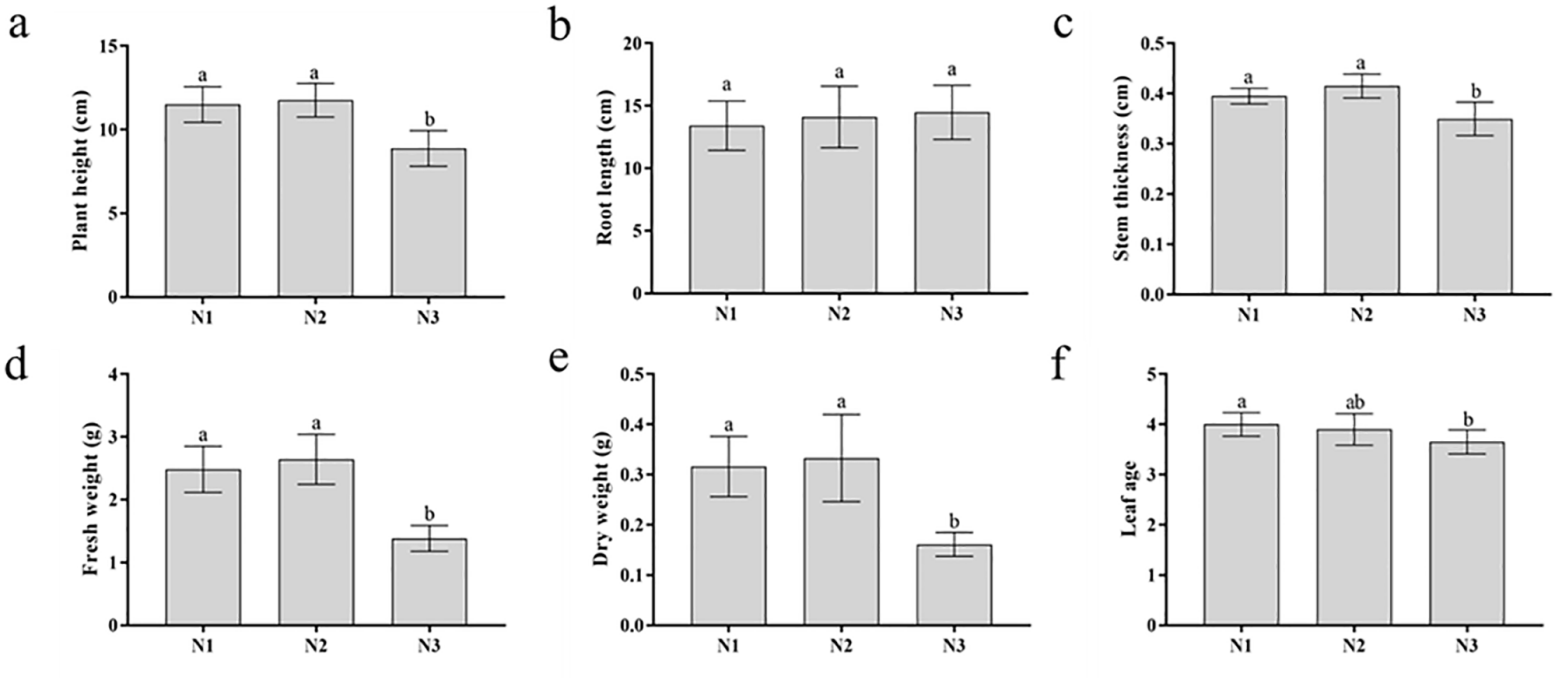
Investigation of watermelon seedling growth parameters under three different nitrogen treatments: N1 (sole nitrate nitrogen), N2 (1:1 mixed nitrate and ammonium nitrogen), and N3 (sole ammonium nitrogen). **(a–f)** represent the statistical results for seedling height, root length, stem diameter, fresh weight, dry weight, and number of true leaves, respectively. Error bars represent the standard errors of the means. Different lowercase letters denote significant differences between treatments, as determined by one-way ANOVA (*P* < 0.05).

The growth parameters of watermelon seedlings, including plant height, root length, stem diameter, fresh weight, dry weight, and number of true leaves, were measured. Seedlings under the N3 treatment displayed significantly lower plant height, stem diameter, fresh weight, dry weight, and number of true leaves compared to those under the N1 and N2 treatments (**Figure 4**).

**Figure 5:**
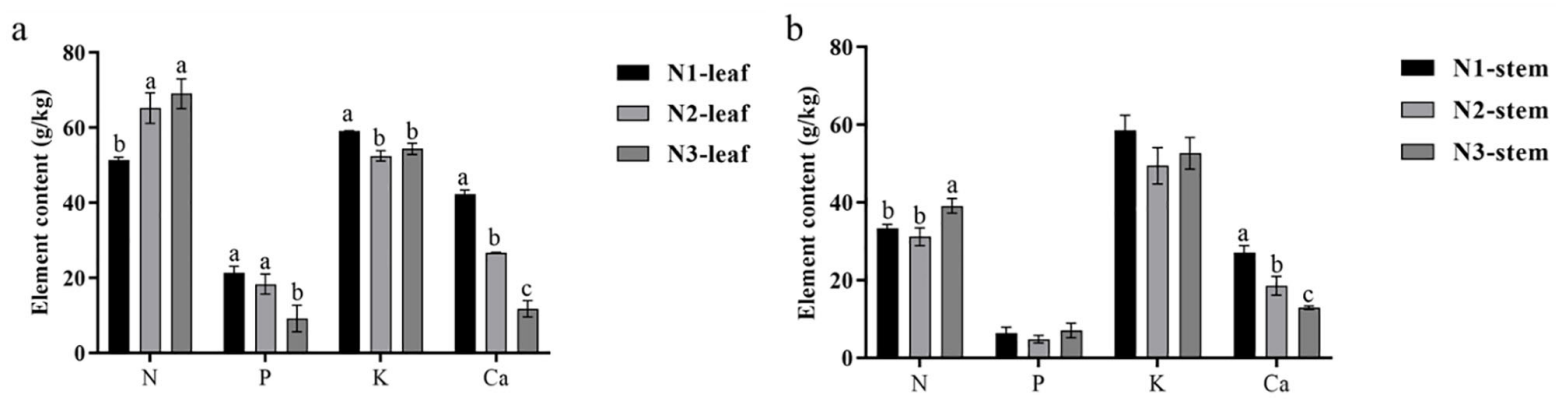
Nitrogen (N), phosphorus (P), potassium (K), and calcium (Ca) contents in watermelon leaves **(a)** and stems **(b)** under three different nitrogen application methods: N1 (sole nitrate nitrogen), N2 (1:1 mixed nitrate and ammonium nitrogen), and N3 (sole ammonium nitrogen). Error bars represent the standard errors of the means. Different lowercase letters indicate significant differences between treatments as determined by one-way ANOVA (*P* < 0.05).

Furthermore, the contents of nitrogen, phosphorus, potassium, and calcium in both leaves and stems were analyzed following treatment with different nitrogen forms. In leaves, nitrogen content under the N1 treatment (51.38 g/kg) was significantly lower than that under the N2 (65.22 g/kg) and N3 (69.08 g/kg) treatments. The phosphorus content in leaves from the N1 and N2 treatments was significantly higher than that under the N3 treatment. Conversely, the potassium (59.13 g/kg) and calcium (42.33 g/kg) contents in leaves were significantly higher under the N1 treatment compared to the N2 and N3 treatments. A similar trend was observed in stems for potassium and calcium contents across the treatments, whereas phosphorus did not show significant variation. Notably, nitrogen content in stems under the N3 treatment was significantly higher than in the other two treatments (**Figure 5**).

### 3.5 Nitrate nitrogen application stimulates cell wall thickening of watermelon sieve tubes

**Figure 6:**
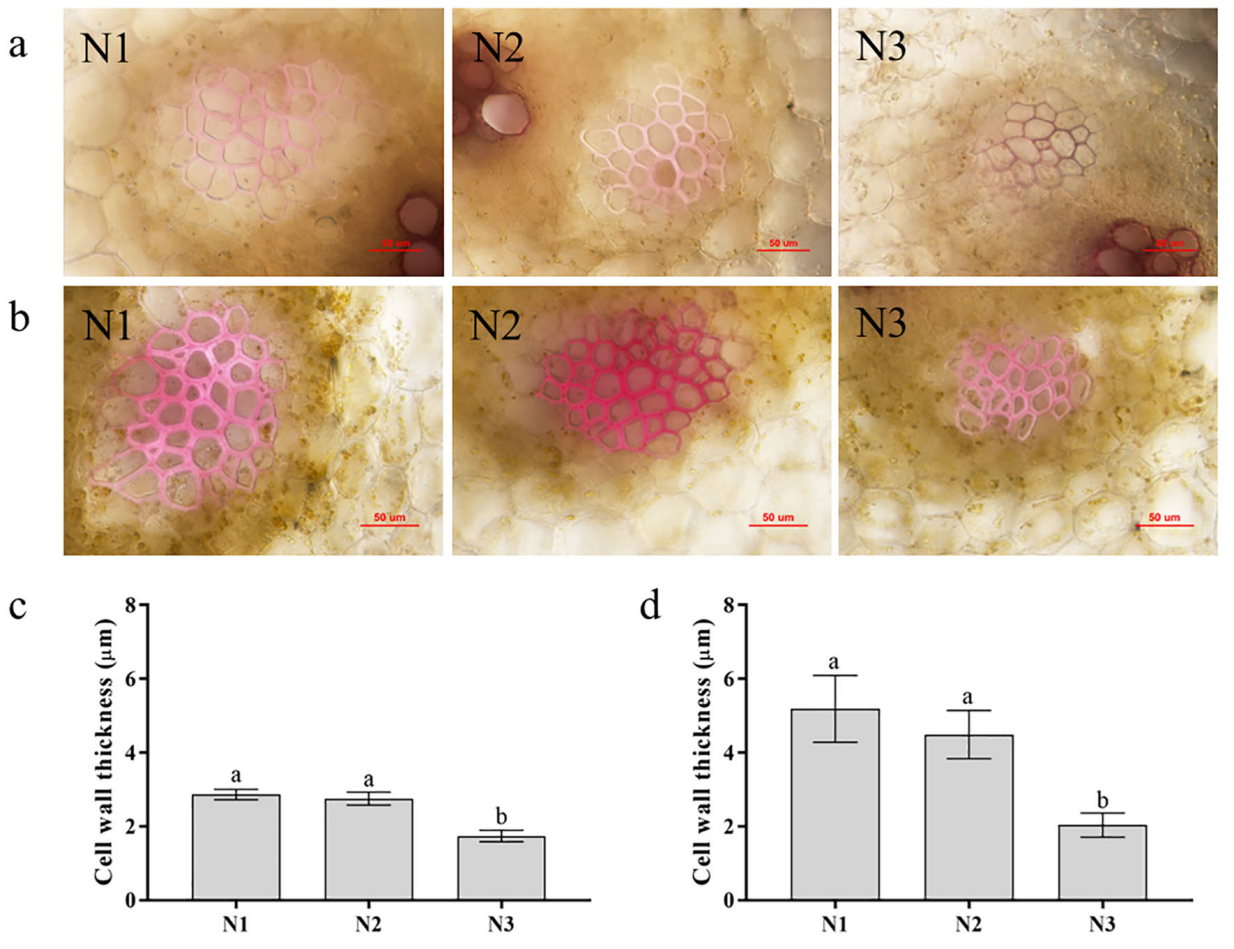
Observation of phloem in watermelon stems. **(a)** Morphology of sieve tubes in non-inoculated watermelon stems under N1 (sole nitrate nitrogen), N2 (1:1 mixed nitrate and ammonium), and N3 (sole ammonium nitrogen); **(b)** Morphology of sieve tubes in watermelon stems inoculated with *A. citrulli* under the three nitrogen treatments; **(c)** Sieve tube cell wall thickness in non-inoculated stems; **(d)** Sieve tube cell wall thickness in inoculated stems. Scale bar = 50 μm. Error bars represent the standard errors of the means. Different lowercase letters indicate significant differences between treatments (one-way ANOVA, *P* < 0.05).

Microscopic measurements showed that the sieve tube cell wall thickness in the phloem of uninoculated watermelon stems was 2.86 μm, 2.75 μm, and 1.74 μm under the N1, N2, and N3 treatments, respectively. Following inoculation with *A. citrulli*, these values increased to 5.19 μm, 4.49 μm, and 2.04 μm, respectively. Notably, the sieve tube cell wall thickness in N3-treated (sole ammonium nitrogen) stems was significantly lower than that in N1- and N2- treated stems (**Figure 6**).

**Figure 7:**
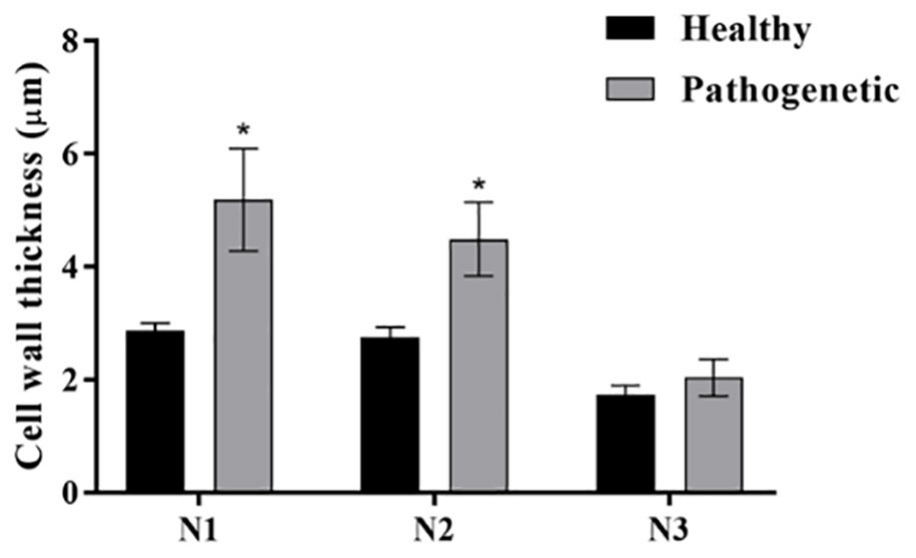
Comparison of sieve tube cell wall thickness in watermelon stems between Aac5-inoculated and non-inoculated plants under three nitrogen treatments: N1 (sole nitrate nitrogen), N2 (1:1 mixed nitrate and ammonium), and N3 (sole ammonium nitrogen). Error bars represent the standard errors, and * indicates a significant difference between inoculated and non-inoculated plants within the same treatment (t-test, *p*  <  0.05).

Further comparative analysis of sieve tube cell wall thickness showed that *A. citrulli* inoculation induced a significant increase in cell wall thickness in N1- and N2- treated seedlings, whereas the increase in N3-treated seedlings was not statistically significant (**Figure 7**).

### 3.6 Nitrogen treatment influences the growth of *A. citrulli* Aac5 in watermelon tissue extracts

**Figure 8:**
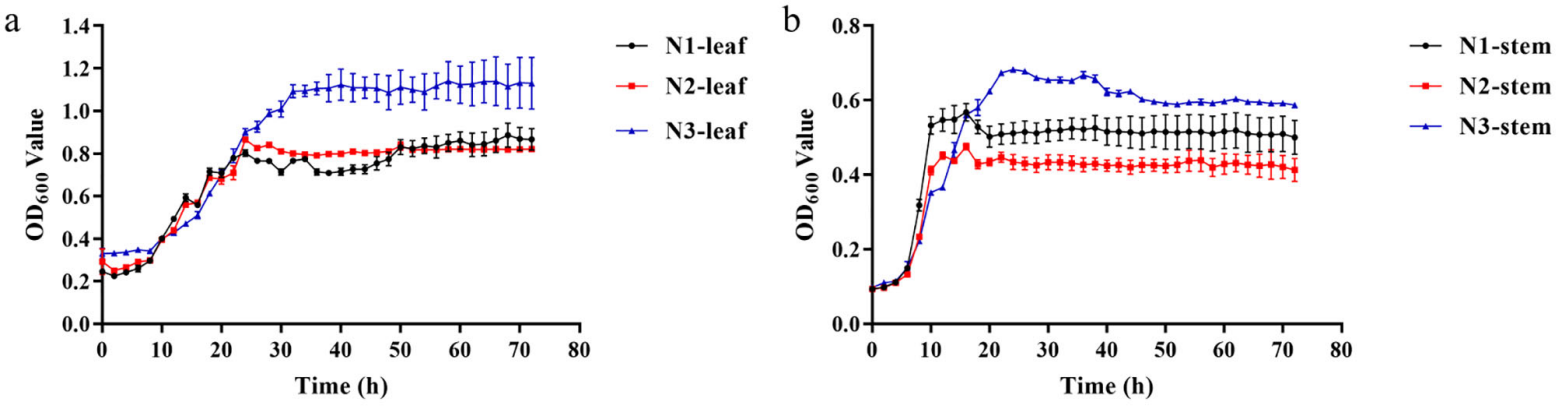
Growth curves of *Acidovorax citrulli* strain Aac5 in leaf **(a)** and stem **(b)** extracts from watermelon seedlings under three nitrogen treatments: N1 (sole nitrate nitrogen), N2 (1:1 mixed nitrate and ammonium nitrogen), and N3 (sole ammonium nitrogen). Bacterial growth was monitored at 28 °C for 96 h with OD_600_ measurements taken every 2 h. Error bars represent the standard errors of the means.

The growth of *A. citrulli* strain Aac5 in both leaf and stem extracts was influenced by the nitrogen treatment applied to the watermelon plants. Initially, bacterial growth in N3-treated tissue extracts was lower than that in N1- and N2- treated extracts. However, this pattern reversed during the later stages of incubation. Specifically, in leaf extracts, Aac5 growth in the N3 treatment was lower in the first 20 h of incubation but exceeded that in the N1 and N2 treatments after 22 h. A similar pattern was observed in stem extracts, where growth under N3 treatment lagged behind until 14 h but exceeded that of the N1 and N2 treatments after 16 h (**Figure 8**).

### 3.7 Analysis of the expression levels of defense-related genes in watermelon

**Figure 9:**
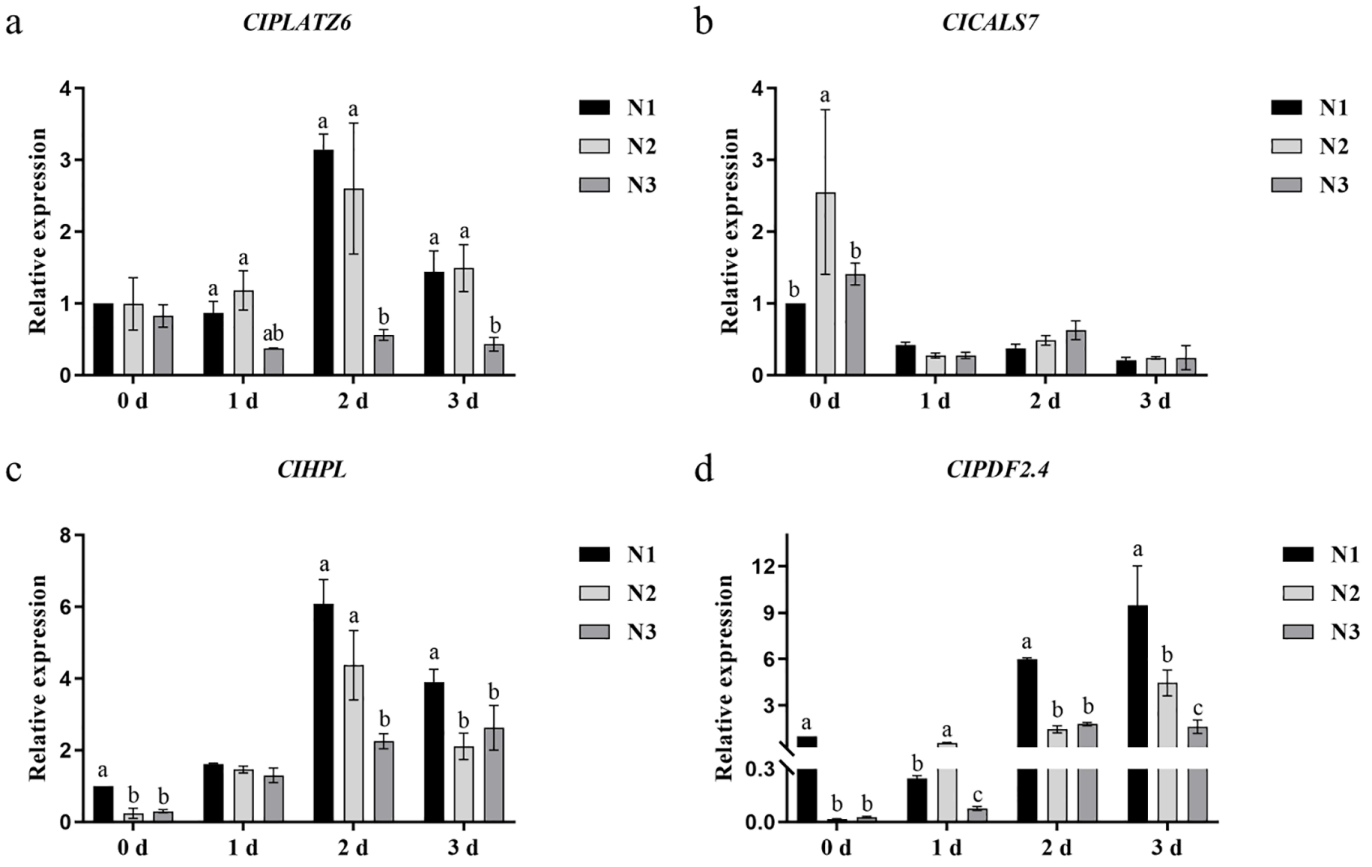
The expression analysis of watermelon defense-related genes, *CIPLATZ6* **(a)**, *CICALS7* **(b)**, *CIHPL* **(c)**, and *CIPDF2.4* **(d)** using *CICAC* as an internal reference gene. N1, N2 and N3 represent different nitrogen fertilizer treatments (sole nitrate nitrogen, 1:1 mixed nitrate and ammonium nitrogen, and sole ammonium nitrogen, respectively). Error bars represent the standard errors of the means.

The results showed that *CIPLATZ6* expression peaked at 2 dpi under the N1 and N2 treatments, and was significantly higher than that under the N3 treatment. The lipid hydroperoxide lyase gene *CIHPL* was upregulated in all watermelon seedlings, with the magnitude of upregulation decreasing as the proportion of nitrate nitrogen in the fertilizer decreased. The expression trend of the defensin-related gene *PDF2.4* in watermelon seedlings was consistent with that of the *CIHPL* gene. The expression level of the callose synthase gene *CICALS7* was downregulated in watermelon seedlings under all nitrogen treatments, with no significant differences in the magnitude of downregulation among the N1, N2, and N3 treatments (**Figure 9**). The results obtained using *CITUA* as the internal reference gene were consistent with those obtained using *CICAC* as the internal reference gene.

### 3.8 Analysis of the expression levels of virulence-related genes in *A. citrulli*

**Figure 10:**
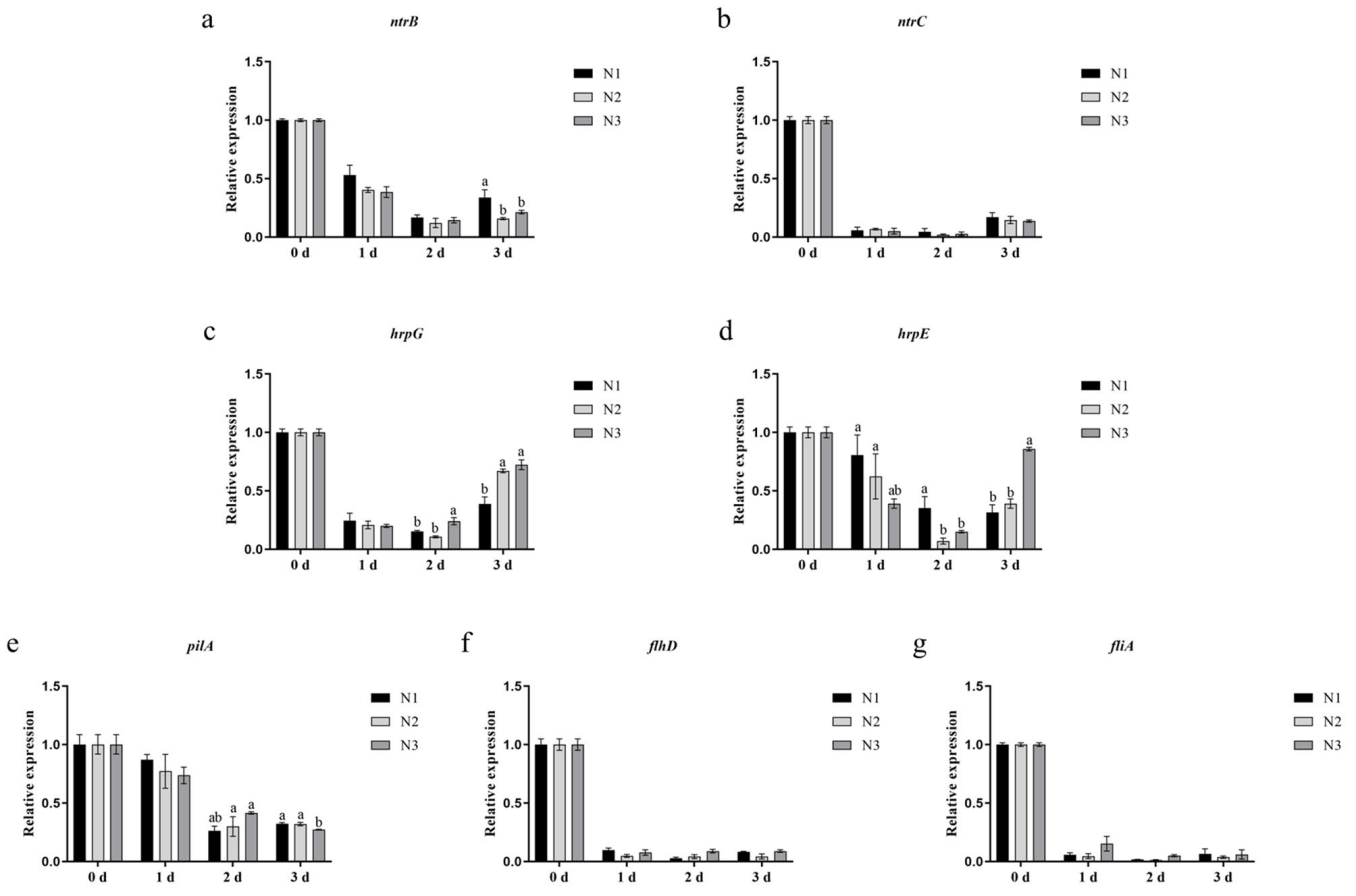
Expression analysis of virulence-related genes [*ntrB* **(a)**, *ntrC* **(b)**, *hrpG* **(c)**, *hrpE* **(d)**, *pilA* **(e)**, *flhD* **(f)**, and *fliA* **(g)**] in Acidovorax citrulli. Treatments N1, N2, and N3 correspond to different nitrogen fertilizer applications: sole nitrate nitrogen, 1:1 mixed nitrate and ammonium nitrogen, and sole ammonium nitrogen, respectively. Error bars represent the standard errors of the means.

The results showed that the expression of all tested *A. citrulli* genes was generally downregulated after inoculation into watermelon cotyledons. Among these, the expression levels of the nitrogen metabolism regulatory genes *ntrB* and *ntrC* decreased to their lowest point at 2 dpi but showed partial recovery by 3 dpi. No significant differences in the expression patterns of *ntrB* and *ntrC* were observed among the different nitrogen fertilizer treatments. Similarly, the expression levels of T3SS-related genes *hrpG* and *hrpE* exhibited an initial decrease followed by an increase. Notably, the expression of these T3SS genes in *A. citrulli* changed more rapidly as the proportion of ammonium nitrogen in the fertilizer increased. In contrast, the pilus-related gene *pilA* and flagella-related genes *flhD* and *fliA* were downregulated after inoculation, with no significant differences in expression among the nitrogen treatments (**Figure 10**).

## 4 Discussion

In this study, we investigated how different nitrogen forms influence watermelon seedling growth, defense responses, and susceptibility to BFB by characterizing plant biomass, stem vascular tissue morphology, expression of defense-related genes, and *A. citrulli* proliferation both *in planta* and in watermelon tissue extracts.

Sole ammonium nitrogen (N3) resulted in a significantly higher disease index than sole nitrate (N1) or a 1:1 nitrate–ammonium mixture (N2). Seedlings under N3 displayed the most severe BFB symptoms, and bacterial populations in stems were markedly higher than in N1. In cotyledons, bacterial populations under N3 were lower during early infection (<96 hpi) but became significantly higher than N1 and N2 after 120 hpi. Similarly, in leaf and stem extracts, *A. citrulli* growth under N3 was initially slower but eventually exceeded that in N1 and N2. These biphasic dynamics indicate that nitrogen form shapes *A. citrulli* colonization in a time- and tissue- dependent manner, rather than exerting a simple promoting or inhibitory effect.

Total nitrogen accumulation was higher in N3-treated seedlings than in N1. We propose that the distinct nitrogen uptake, transport, and assimilation patterns between nitrate and ammonium underlie these pathogen dynamics. Nitrate is readily absorbed and translocated to shoots, supporting rapid early pathogen proliferation. By contrast, ammonium uptake is energy-demanding and poorly mobile (Xu et al., 2012), causing mild physiological stress and slower initial colonization; yet the higher total nitrogen in ammonium-fed plants creates a nutrient-rich environment that favors sustained late-stage pathogen growth. Thus, the observed patterns cannot be attributed solely to host resistance, but involve complex nutritional interactions between the host and pathogen (Feng et al., 2026). Notably, N3 seedlings exhibited significantly reduced growth, including lower plant height, stem diameter, biomass, and leaf number, indicating physiological stress or mild ammonium toxicity. This weakened vigor likely contributed to elevated disease severity, independent of specific defense responses.

Nitrate nitrogen had a more positive effect on promoting watermelon seedling growth. Measurement of calcium levels in stems and leaves under each treatment showed a significant increase in calcium content with higher nitrate nitrogen application. Given that calcium is a critical nutrient for plant defense, increased calcium accumulation may enhance watermelon resistance to BFB (Wang et al., 2017). Additionally, histological analysis of stem tissues indicated that nitrate nitrogen application promoted the development of watermelon stem vascular tissue, resulting in thicker sieve tube cell walls. In contrast, *A. citrulli* inoculation did not induce a significant increase in sieve tube cell wall thickness in stems treated with ammonium nitrogen alone.

Expression analysis of watermelon defense genes showed that *CIHPL* and *CIPDF2*.4 were upregulated after inoculation, most strongly in N1. In contrast, *CIPLATZ6* was significantly upregulated in N1 and N2 but unchanged in N3, implying that nitrate specifically regulates this gene during *A. citrulli* infection. The expression pattern of *CIHPL* in watermelon differed from its downregulation in melon (Zimerman-Lax et al., 2016), likely due to host species differences or reference gene selection (Kong et al., 2017). Meanwhile, *CICALS7* was downregulated in all treatments, possibly due to transcriptional suppression by the pathogen.

Analysis of virulence-related gene expression in *A. citrulli* showed that most tested genes were downregulated following inoculation into watermelon cotyledons. In a previous study, inoculation of *A. citrulli* strain FC440 into yellow melon cotyledons caused no obvious changes in the expression of T3SS regulatory genes at 48 dpi, but significantly suppressed genes involved in quorum sensing and chemotaxis (Youlituzi et al., 2021). The differential expression patterns of T3SS genes observed in the present study might be attributed to differences between the two *A. citrulli* strains FC440 and Aac5, which belong to distinct genetic groups. Furthermore, the previous study only examined gene expression at a single time point (48 hpi), providing an incomplete characterization of the temporal changes in T3SS-related gene expression during infection. In the present study, the expression of T3SS genes *hrpG* and *hrpE* showed an initial decrease followed by an increase. Notably, the expression changes of *hrpG* and *hrpE* occurred more rapidly with increasing proportions of ammonium nitrogen in the fertilizer. These results indicate that ammonium nutrition facilitates faster activation of virulence-related genes and accelerates the infection process of *A. citrulli*, or nitrate nitrogen delays or inhibits this process. In contrast, genes associated with nitrogen metabolism regulation (*ntrB* and *ntrC*), pili (*pilA*) and flagella (*flhD* and *fliA*) were downregulated after inoculation, with no significant differences among the three nitrogen fertilizer treatments.

This study has limitations that warrant caution in interpretation. The nutrient solutions differed not only in nitrogen form but also in accompanying ions such as chloride and sulfate, which may have influenced plant and pathogen responses. Furthermore, the controlled environment, and soilless culture system differ from field conditions, limiting direct practical extrapolation. Some experimental replications were relatively limited, which may reduce statistical robustness. Despite these constraints, our findings remain meaningful for soilless and substrate-based watermelon production systems, where nitrogen form management is increasingly important.

In summary, nitrogen form strongly influences BFB severity in watermelon by modulating seedling vigor, physical defense, nutrient status, defense gene expression, and pathogen infection dynamics. Nitrate mitigates disease severity, while sole ammonium exacerbates it through combined effects of weakened plant growth, altered tissue nutrition, and accelerated pathogenicity activation. This work provides a theoretical basis for nutrient management strategies to suppress BFB in watermelon production.

## Data Availability

The original contributions presented in the study are included in the article/supplementary material. Further inquiries can be directed to the corresponding authors.
